# *DMP1* C-Terminal Mutant Mice Recapture the Human ARHR Tooth Phenotype

**DOI:** 10.1002/jbmr.117

**Published:** 2010-04-30

**Authors:** Baichun Jiang, Zhengguo Cao, Yongbo Lu, Carol Janik, Stephanie Lauziere, Yixia Xie, Anne Poliard, Chunlin Qin, Leanne M Ward, Jian Q Feng

**Affiliations:** 1 Institute of Medical Genetics, Shandong University School of Medicine Jinan, People's Republic of China; 2 Department of Biomedical Sciences, Baylor College of Dentistry Dallas, TX, USA; 3 The State Key Laboratory Breeding Base of Basic Science of Stomatology (Hubei-MOST) & Key Laboratory of Oral Biomedicine Ministry of Education, School & Hospital of Stomatology, Wuhan University,Wuhan People's Republic of China; 4 Departments of Pediatrics and Surgery, Children's Hospital of Eastern Ontario, University of Ottawa Ottawa, Ontario, Canada; 5 Inserm UMR S747, Université Paris Descartes 45 rue des Saints-Pères 75006 Paris France

**Keywords:** *DMP1*, ARHR, odontoblast, FGF-23, hypophosphatemia

## Abstract

*DMP1* mutations in autosomal recessive hypophosphatemic rickets (ARHR) patients and mice lacking *Dmp1* display an overlapping pathophysiology, such as hypophosphatemia. However, subtle differences exist between the mouse model and human ARHR patients. These differences could be due to a species specificity of human versus mouse, or it may be that the mutant *DMP1* in humans maintains partial function of DMP1. In this study we report a deformed tooth phenotype in a human *DMP1* deletion mutation case. Unexpectedly, the deletion of nucleotides 1484 to 1490 (c.1484_1490delCTATCAC, delMut, resulting in replacement of the last 18 residues with 33 random amino acids) showed a severe dentin and enamel defect similar to a dentinogenesis imperfecta (DI) III–like phenotype. To address the molecular mechanism behind this phenotype, we generated delMut transgenic mice with the endogenous *Dmp1* gene removed. These mutant mice did not recapture the abnormal phenotype observed in the human patient but displayed a mild rachitic tooth phenotype in comparison with that in the *Dmp1-*null mice, suggesting that the DI III–like phenotype may be due to an as-yet-undetermined acquired gene modifier. The mechanism studies showed that the mutant fragment maintains partial function of DMP1 such as stimulating MAP kinase signaling in vitro. Last, the in vitro and in vivo data support a role of odontoblasts in the control of fibroblast growth factor 23 (FGF-23) regulation during early postnatal development, although this regulation on P_*i*_ homeostasis is likely limited. © 2010 American Society for Bone and Mineral Research.

## Introduction

Mutations in fibroblast growth factor 23 (*FGF23)* and *PHEX* account for approximately 60% of hypophosphatemic rickets with eucalciuria and are associated with abnormalities in dentin and bone.(1) Mutations in the type 2a sodium-phosphate cotransporter lead to hereditary hypophosphatemic rickets with hypercalciuria (HHRH).(2–6) The molecular defects responsible for the remaining hypophosphatemic patients are unknown.

Previously, we reported that *Dmp1-*null newborns displayed no gross phenotype(7) but developed hypophosphatemia including hypomineralized bone,(8) defects in chondrogenesis,(9) partial failure of predentin maturation into dentin, and expansion of the pulp cavities and root canals during postnatal tooth development.(10) These data support a dual role of DMP1 in both local and systemic regulation of phosphate (P_*i*_) homeostasis.(11,12)

We and others also reported that *DMP1* mutations are responsible for autosomal recessive hypophosphatemic rickets (ARHR) in human patients, including bone defects and an increase in fibroblast growth factor 23 (FGF-23) levels (although some of *DMP1* mutation patients overlapped with the normal range for FGF-23).(12–14) However, there is a phenotypic difference between human patients (mild) and *Dmp1-*null mice (severe). These differences could be due to a species specificity of human versus mouse or to the fact that the mutant fragment may maintain partial DMP1 function. Furthermore, few clinical cases of ARHR have been reported so far; therefore, it is challenging to obtain human cases for pathophysiologic studies.(12,13,15) Thus generation of *DMP1* mutant mouse models based on the human mutations is critical not only for distinguishing these differences but also for molecular pathophysiologic studies of this disorder.

The tooth phenotype in our previously reported ARHR cases(12) has not been reported. DMP1, highly expressed in odontoblast(7) and osteocyte cells,(16) is cleaved into N-terminal and C-terminal fragments,(11) but it is not clear which fragment is functionally critical for bone and tooth formation both in vivo and in vitro.

The goals of this study were (1) to report the *DMP1* mutant tooth phenotype from one of our previously reported clinical cases,(12) (2) to determine the molecular genetics and pathophysiology of *DMP1* mutations through generation and analysis of a mouse model harboring the *DMP1* deletion mutation compared with *Dmp1-*null mice, (3) to understand how the *Dmp1* mutant changes cell signaling in vitro, (4) to determine whether *Dmp1-*null or *Dmp1* delMut mice develop an enamel phenotype, and (5) to test whether odontoblast cells play a role in control of *FGF23* expression.

## Materials and Methods

### Human subjects

Dental radiographs were obtained from one individual with ARHR owing to the delMut described previously (patient F_1-2_).(12) Patient F_1-2_ had unique clinical features that distinguished her from the other hypophosphatemic patients in these kindreds, including global developmental delay and a seizure disorder. To date, it remains unclear whether these additional features are linked to the delMut in *DMP1*; however, their presence in a single patient who has two affected siblings without these features raises the likelihood that they are not part of the ARHR phenotype.

### Generation of mutant *Dmp1* transgenic mice

A full-length mouse *Dmp1* cDNA was cloned, and 7 nucleotides corresponding to the 1484-1490del mutation of human *DMP1* in ARHR patients(12) were deleted by site-directed mutagenesis using the QuikChange II Site-Directed Mutagenesis Kit (Stratagene, La Jolla, CA, USA) with primers DMUT-F (GAAACTAATAGTTGATGCAACAAA CCCATTGGGGACCAAGATG) and DMUT-R (CATCTTGGTCCCCAATGGGTTTGTTGCATCAACTATTAGTTTC). For generation of a mutant *Dmp1* transgene, the full-length mouse *Dmp1* cDNA with the 7-nucleotide deletion and an SV40 polyadenylation signal were cloned into a mammalian expression vector(17) (graciously provided by B Kream and A Lichtler, University of Connecticut Health Center, Farmington, CT, USA) containing the 3.6-kb rat type I collagen promoter plus a 1.6-kb intron 1 (*Col1a1* promoter) that is highly active in pulp cells, odontoblasts, and osteoblasts, giving rise to the *Col1a1-*mutant *Dmp1* transgene. The transgene was released by SacII and SalI from the vector backbone and purified for pronuclear injection. Transgenic founders with CD1 background were generated. Three of five independent transgenic lines were used for crossing to *Dmp1-*null mice (see below).

### Target expression of the mutant *Dmp1* transgene in *Dmp1-*null mice

We have previously described the generation of *Dmp1-*null mice using the *lacZ* knock-in targeting approach.(7) For expression of the mutant *Dmp1* transgene in *Dmp1-*null mice, *Col1a1-*mutant *Dmp1* transgenic mice were first crossed with heterozygous (HET) *Dmp1-*null mice to generate HET *Dmp1-*null mice with the *Col1a1-*mutant *Dmp1* transgene (*Tg*^+/–^;*Dmp*1^+/–^). These mice were further bred with homozygous *Dmp1*-null mice to generate homozygous *Dmp1-*null mice carrying the mutant *Dmp1* transgene (Tg^+/–^;*Dmp*1^–/–^). This mouse line is designated *mutant mouse* (Mut). Since no phenotypic differences between the wild-type and heterozygous *Dmp1-*null mice were found.(9,10) the littermate HET *Dmp1* mice were used for control.

The two *Dmp1* transgenic mouse lines(18) *Col1a1P-Dmp1* transgene (under the control of the same promoter as used for driving the mutant *Dmp1* transgene) and *DsppP-Dmp1* transgene (under control of the 6-kb mouse *Dspp* promoter, actively expressed only in late odontoblast cells)(19) were crossed into *Dmp1-*null mice.(7) Samples of three developmental stages (10 days old, 3 weeks old, and 2 months old) were obtained and analyzed for this study. All mice were bred to a CD1 background, an outbred strain for getting closer to the physiologic condition. The animal research has complied with all relevant federal guidelines and institutional policies.

### PCR genotyping

Genomic DNA was extracted from a tail biopsy and used for genotyping by PCR analysis. Primers p01 (5'-GAGTGCGATCTTCCTGAGGCCGATACTGTC-3') and p02 (5'-CGCGGCTGAAATCATCATTAAAGCGAGTGG-3') were used for detection of the *Dmp1-*null allele (490 bp), and primers p03 (5'-GCCCCTGGACACTGACCATAGC-3') and p04 (5'-CTGTTCCTCACTCTCACTGTCC-3') were used for detection of the wild-type allele (421 bp). The primers p05 (5'-CAGCCGTTCTGAGGAAGACAGTG-3' from *Dmp1* cDNA) and p06 (5'-TGTCCAAACTCATCAATGTATCT-3' from the SV40 polyadenylation signal) were used for detection of the wilt-type (WT) or mutant *Dmp1* transgene (337 or 330 bp, respectively).

### High-resolution tooth radiography and micro–computed tomography (µCT)

The mandible samples from control, *Dmp1*-null, and mutant mice were dissected free of muscle, and the first molars of the maxilla were extracted as described previously.(10) Briefly, fresh mandibles were incubated in lysis buffer (2× SSC, 0.2% SDS, 10 mM EDTA, and 1 mg/mL proteinase K) for 1 to 2 days at 55 °C. After the muscles surrounding teeth were digested, the molars were extracted under a dissection microscope. The intact mandibles and first molars then were examined radiographically using a high-resolution radiography system (piXarray 100, Micro Photonics, Allentown, PA, USA). µCT analyses include a low-resolution scan of one hemimandible per animal for overall assessment of shape and structure and a high-resolution (6-µm) scan of a more limited anatomic area (200 slices) in a specified region of interest for detailed 3D morphometric analyses using a Scanco µCT 35 (Scanco Medical AG, Brüttisellen, Switzerland).

### Histologic preparation

Mandible specimens from control, *Dmp1-*null, mutant, and rescued mice were dissected and fixed in 4% paraformaldehyde at 4°C overnight, followed by two different processes: (1) One side specimens were decalcified, dehydrated, and embedded in paraffin and then sectioned for in situ hybridization, hematoxylin and eosin (H&E) staining, and safranin-O staining, and (2) the other side specimens were dehydrated and embedded in methyl methacrylate, and nondecalcified sections were used for dentin formation rate and scanning electron microscopy (SEM).

### In situ hybridization

The mouse antisense RNAs for *Dmp1, Fgf23,* and *Phex* were used for in situ hybridization, as described previously.(16) Briefly, digoxigenin (DIG)–labeled mouse cRNA probes were prepared by using an RNA Labeling Kit (Roche, Indianapolis, IN, USA). The hybridization temperature was set at 55°C, and the washing temperature was set at 70°C so that endogenous alkaline phosphatase was inactivated. DIG-labeled nucleic acids were detected in an enzyme-linked immunoassay with a specific anti-DIG-AP antibody conjugate and an improved substrate that gives rise to a red signal (Vector, Burlingame, CA, USA) according to the manufacturer's instructions.

### Double fluorochrome labeling

To examine the dentin formation rate, double fluorescence labeling was performed as described previously.(12,18) Briefly, an alizarin red label (20 mg/kg i.p.; Sigma-Aldrich, St. Louis, MO, USA) was administered to 12-day-old mice, followed by administration of a calcein label (5 mg/kg i.p.; Sigma-Aldrich) 7 days later. Mice were euthanized 2 days after the second injection (3 weeks old). The nondecalcified upper mandibles were sectioned, and unstained sections were photographed under epifluorescence illumination using a Nikon E800 microscope (Nikon Instruments, Melville, NY, USA).

### Generation of recombinant DMP1 proteins, cell cultures, stains-all staining, and Western blot

The full-length *DMP1*, C-terminal *DMP1*, and full-length delMut *DMP1*cDNAs were cloned originally into the pcDNA3 vector.(20) The empty pcDNA3 vector, pcDNA3-WT *DMP1* vector, and pcDNA3-delMut *DMP1* vector, were transfected into 293 cell lines for 24 hours, followed by serum-free medium culture for another 24 hours for confirming their secretion in the medium. Then 5 µL of condition medium was loaded to 4% to 20% gradient polyacrylamide gels, followed by staining with stains-all staining agent, as described previously.(21)

The full-length *DMP1*, C-terminal *DMP1*, and full-length delMut *DMP1* cDNAs were released from the pcDNA3 vector and r-cloned into the PGEX4T-2 vector separately. These vectors then were transformed into the bacterial host BL21 for production of recombinant DMP1-Glutathione S-transferase fusion proteins, followed by removal the GST tag using thrombin (Sigma-Aldrich) according to the manufacturer's recommendations. For testing the effects of these recombinant DMP1s on MAPK signaling, an odontoblast cell line(22) was grown to 70% confluency. Cells were serum-deprived for 16 hours prior to recombinant DMP1 treatment. Cells were treated in triplicate with 250 ng/mL of full-length, C-terminal, or the delMut DMP1 and harvested at different time points (0, 5, 10, 30, and 60 minutes) for MAPK activation. The concentration of cell lysates was determined by using the BCA kit (Pierce, Rockford, IL, USA). Then 10 µg of total protein was subjected to 10% SDS-PAGE gel, followed by blotting onto polyvinylidene difluoride membranes, and probed with the following antibodies: anti-phosphorylated-ERK (p-ERK), ERK, and β-actin (used at 1:1000 dilution; Cell Signaling Technology, Danvers, MA, USA). The Western blot was performed using chemiluminescence (Perkin Elmer, Waltham, MA, USA) of horseradish peroxidase (HRP) linked to a second antibody (Cell Signaling, Inc.). The membranes then were exposed to X-Omat Kodak film (Perkin Elmer) to visualize the bands.

### Real-time RT-PCR

Newborn primary calvarial and pulp cells were isolated and cultured for 48 hours, followed by real-time RT-PCR for quantitation of *Fgf23* mRNA. Briefly, total RNA was extracted by using a Trizol reagent (Invitrogen, Carlsbad, CA, USA). Freshly isolated RNA was reverse transcribed with a Super Script first-strand synthesis system (Invitrogen) following the manufacturer's recommendations. The resulting cDNA then was subjected to quantitative real-time PCR using 2× Brilliant SYBR QPCR Green Master Mix Reagent (Stratagene, Cedar Creek, TX, USA). The sequences of the primers were for *Fgf23*, 5'-TACTTGTCGCAGAAGCATCAC-3' and 5'-GGCGAACAGTGTAGAAATGCAG-3', and for *Gapdh*, 5'-ACCACAGTCCATGCCATCAC-3' and 5'-TCCACCACCCTGTTGCTGTA-3'. Three independent measurements per sample were performed. The quantified individual RNA expression levels were normalized to *Gapdh*.

### Serum biochemistry

Ten-day-, 3-week-, and 2-month-old male mice were anesthetized, blood was collected though heart puncture, and serum samples were obtained by precipitation and centrifugation. Serum P_*i*_ levels were measured using a Phosphorus Liqui-UV Kit (Stanbio Laboratory, Boerne, TX, USA). Serum calcium levels were assayed using a Calcium Liquicolor Kit (Stanbio Laboratory) following the manufacturer's protocol. Serum intact FGF-23 levels were measured using an FGF-23 ELISA Kit (Kainos Laboratories, Tokyo, Japan), as described previously.(12)

### Statistical analysis

Data were expressed as the mean ± SEM. Data were evaluated statistically by ANOVA to test for any differences among the three groups of mice. If significant differences were found by ANOVA, the Bonferroni method of multiple comparisons was used to determine which groups were significantly different from each other. A *p* value of less than .05 is considered statistically significant.

## Results

### *DMP1* deletion mutation led to DI III–like tooth phenotype

**Fig. 1:**
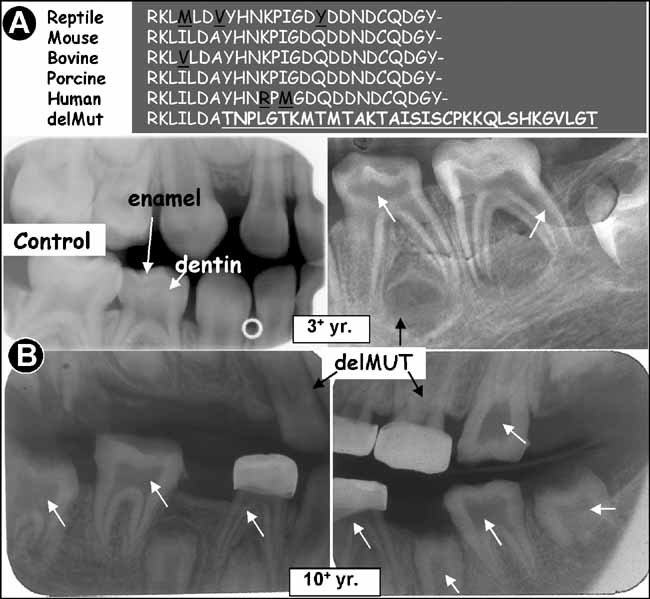
Tooth phenotypes in a delMut ARHR patient. (*A*) A schematic amino acid comparison of the end of the C-terminal region of the *DMP1* gene among different species, including the 1484-1490del mutation previously reported.(12) The 1484-1490del mutation resulted in a frameshift that replaced the conserved C-terminal 18 residues with 33 nonnative residues. (*B*) Radiographs from a 3-year-old 1484-1490del mutation patient (*upper right panel*) and an age-matched control (*upper left panel*), as well as radiographs from the same patient at 10 years of age (*lower panels*). Changes in tooth shape, severe reduction in dentin thickness, large pulp/root canals, and loss of enamel are shown in both primary and permanent teeth (*arrows*).

Previously, we reported that the deletion of nucleotides 1484 to 1490 of *DMP1* (delMut) resulted in the last 18 amino acids being replaced by 33 nonnative residues(12) (Fig. 1*A*). A radiograph from a delMut child at age 3+ showed significantly enlarged pulps and root canals with extremely thin dentin and loss of enamel (Fig. 1*B*, *upper right panel, white arrows*) in comparison with an age-matched control child (Fig. 1*B*, *upper left panel*). At age 10, the same child showed a DI III–like phenotype, including the following features: lack of well-mineralized dental enamel, enlarged pulp chamber, and enlarged root canal, which are likely a result of decreased dentin formation as well as malformed tooth shape (Fig. 1*B*, *lower panels*).

The severe tooth phenotype, affecting both dentin and enamel, in the delMut patient raised the following questions: Is the tooth phenotype a primary consequence of the *DMP1* mutation or the result of a dominant-negative role of this mutant or other events trigged by the *DMP1* mutant? The simplest way to address this dilemma is to generate a delMut mouse model mimicking the same mutation observed in human patients and to test whether this deletion mutation will recapture the same tooth phenotype in mice.

### Overexpression of delMut transgene driven by a 3.6-kb *Col1a1* promoter in a wild-type background has no apparent phenotype

**Fig. 2:**
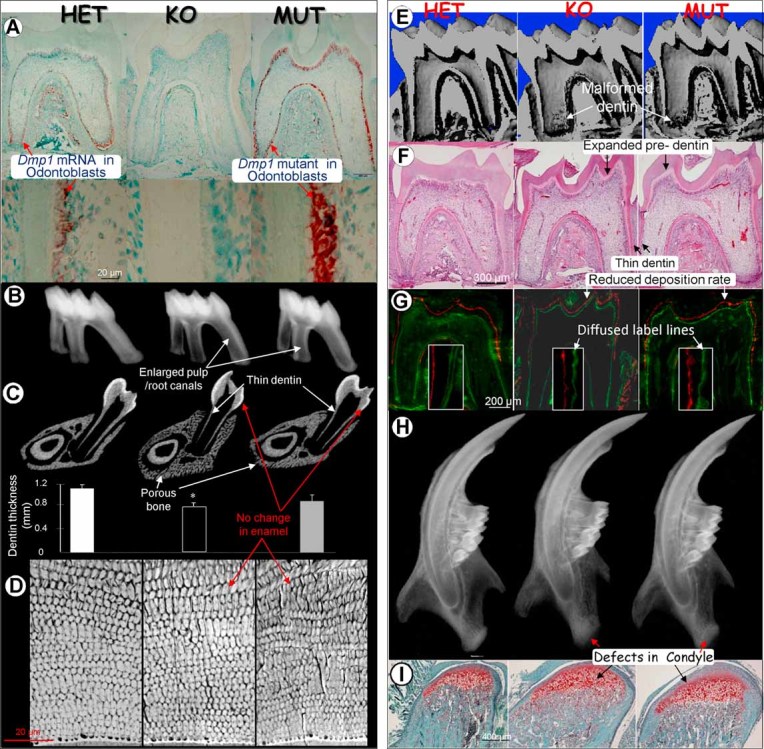
The delMut *Dmp1* recaptures the mild *Dmp1-*null dentin phenotype with no change in enamel. (*A*) In situ hybridization (red staining with green nuclear counterstain) for *Dmp1* in the lower first molar in 10-day-old mice. In the heterozygous control (HET), *Dmp1* is expressed in odontoblasts (*left panels*). In *Dmp1* knockout (KO) mice, there is no *Dmp1* signal (*middle panels*). In *Dmp1-*null mice with the deletion mutant (MUT) full cDNA reexpressed in odontoblasts (under control of the 3.6-kb *Col1a1* promoter), the MUT expression level is very high (*right panels*). (*B*) Radiographs of the first upper molars of HET (*left*), KO (*center*), and MUT (*right*) mice, with enlarged pulp/root canals observed in KO and mutant teeth. (*C*) Coronal section µCT views obtained from the first lower molars of HET (*upper left*), KO (*upper center*), and MUT (*upper right*) mice, exhibiting thin dentin and porous alveolar bone in KO (more striking) and MUT (milder) teeth. Quantitative µCT data (*lower panel*) reveal a significant difference between HET and NULL groups (*p < .05 by Student's *t* test, *n* = 4). The MUT group shows the same trend but with no significance compared with the HET group. (*D*) Backscattered SEM images show that there is no an apparent difference in the enamel prism structure and distribution among control (*left*), KO (*center*), and MUT (*right*) groups. (*E*) 3D µCT images of the first^t^ lower molars obtained from these three groups, revealing porous dentin structures in the KO (*center*) and mutant (*right*) mice. (*F*) H&E stains of the first lower molars, revealing a thin dentin layer and an enlarged predentin layer in KO (*center*) and MUT (*right*) mice. (*G*) Fluorochrome double-labeled sections of the lower first molars, revealing reduction of the dentin mineralization rate plus diffused labeling lines in KO (*center*) and MUT (*right*) mice. (*H, I*) The abnormalities of condyle formation in KO (*center*) and MUT (*right*) mice as documented with radiographs (*F*) and safranin O assays (*I*).

To generate a delMut mouse model harboring the human *DMP1* 1484-1490del mutation, we first generated a transgenic mouse harboring a full-length *Dmp1* cDNA with the same deletion mutation as observed in the patients under the control of a 3.6-kb *Col1a1* promoter, which is highly active in pulp cells, odontoblasts, and osteoblasts.(17,23) Previously, we have successfully rescued the *Dmp1-*null tooth phenotypes using the full-length *Dmp1* cDNA under the control of the same promoter(18); thus this promoter might have the proper spatial-temporal characteristics to mimic the endogenous *Dmp1* gene expression in teeth. Five independent *Col1a1*-mutant *Dmp1* transgenic lines were obtained, and three independent lines were used for this study. These three lines (2, 11, and 21) have different expression levels (see the RT-PCR data in Supplemental Fig. 1), although none of these lines shows an apparent phenotype (data not shown), excluding a dominant-negative role of this mutant in the WT background. In addition, all three lines in the *Dmp1-*null background show an identical expression pattern, although only line 2 is shown in Fig. 2*A*.

### *Dmp1* mutant mice display abnormalities in dentinogenesis and condyle formation with little change in enamel

Next, we crossed these three independent lines of the delMut mice into the *Dmp1-*null background. All these independent lines display an identical dentin-defect phenotype (see below), although only one of the mutant lines is presented (line 2). Note that we have previously reported that *Dmp1-*null mice displayed a partial failure of maturation of predentin into dentin, hypomineralization of the dentin, and expansion of the pulp cavities and root canals during postnatal tooth development.(10)

To determine whether the *Dmp1* delMut mice display the same phenotype as the mutant individual, the mutant (Fig. 2, *right panels*), the *Dmp1-*null (Fig. 2, *middle panels*), and the control mice (Fig. 2, *left panels*) were examined by a combination of radiography, micro–computed tomography (µCT), histology, and a double-labeling assay, as well as backscattered SEM. Like *Dmp1-*null mice, *Dmp1* mutant mice showed a thin dentin and an enlarged pulp cavity and root canal with radiographic and µCT analyses (Fig. 2*B, C*). Note that the quantitative analysis showed a significant reduction of dentin in the *Dmp1-*null mice, with *p* < .05 compared with the control, whereas the reduction in dentin in the mutant is not significantly different from control (Fig. 2*C*, *lower panel*).

Importantly, both the µCT image (Fig. 2*C*) and backscattered SEM images (Fig. 2*D*) showed identical mineral density and prism structure in control, knockout, and mutant mice. High-resolution 3D µCT images showed that the root dentin is porous in the root canals of both the *Dmp1-*null and delMut mice (Fig. 2*E*). H&E staining showed similar changes: expanded predentin and thin dentin in both *Dmp1-*null and *Dmp1* mutant mice compared with control mice (Fig. 2*F*). The double-labeling assay also showed similar reductions in dentin formation rate and diffused labeling lines in both *Dmp1-*null and *Dmp1* mutant mice (Fig. 2*G*). Lastly, both *Dmp1-*null and mutant mice showed similar defects in condyle formation, with expanded hypertrophic chondrocytes in condyles (Fig. 2*H, I*).

Taken together, all the assays just described showed similar defects in both *Dmp1-*null and mutant mice, supporting a hypothesis that the delMut *DMP1*–targeted transgene recaptures the *Dmp1-*null dentin phenotype without an apparent sign of enamel defects, although the mutant phenotype is slightly milder than that of null mice.

### The delMut *DMP1*–targeted transgene recaptures the mild changes in serum FGF-23 and P_*i*_ levels

**Fig. 3:**
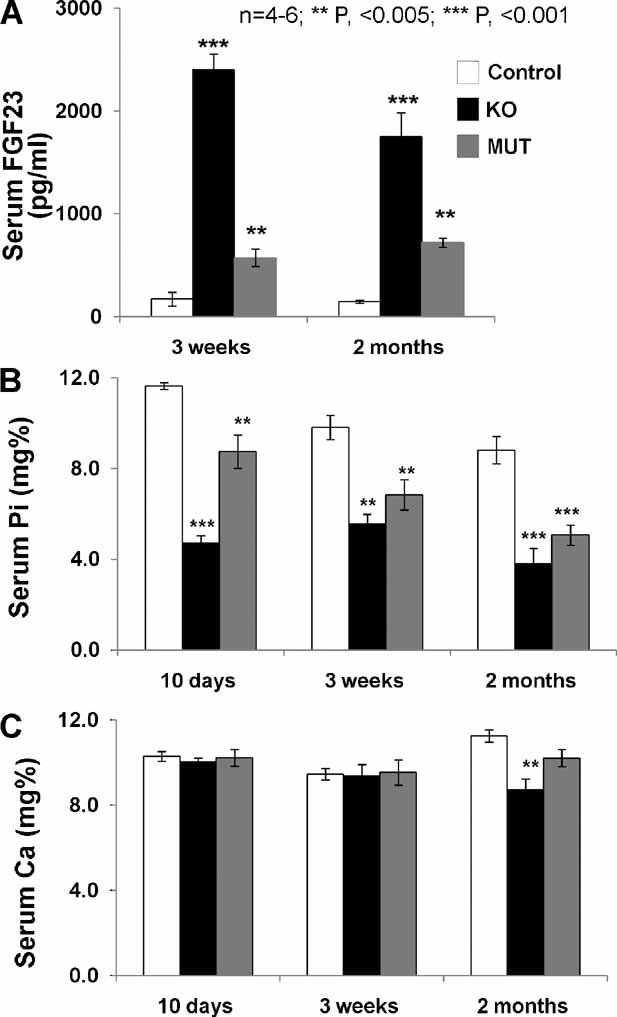
Serum changes in FGF-23, P_*i*_, and Ca in *Dmp1-*null and delMut mice. (*A*) Changes in serum FGF-23 at 3 weeks and 2 months of age. (*B*) Changes in serum P_*i*_ at 10 days, 3 weeks, and 2 months of age. (*C*) No apparent changes in serum Ca at 10 days, 3 weeks, and 2 months of age except a minor change in *Dmp1* KO mice at 2 months.

We previously reported that delMut patients display mild changes in FGF-23 (<2-fold changes) and P_*i*_ levels (<20% reduction) in comparison with the normal controls.(12) In this study we showed a mild increase in FGF-23 (2- to 3-fold) in delMut mice compared with control (Fig. 3*A*). Similarly, the change in P_*i*_ level also was mild in all three age groups tested (Fig. 3*B*). As predicted, the serum calcium level is largely unchanged in both *Dmp1-*null and delMut groups (Fig. 3*C*). These data suggest that the delMut *DMP1* has a partial function of DMP1.

### Roles of DMP1 recombinant proteins in activation of MAPK signaling vary

Recently, Wu and colleagues (http://iadr.confex.com/iadr/2008Dallas/techprogram/abstract_101678.htm) showed that DMP1 targeted to αvβ-integrin and activated the MAPK-Erk and Jnk-AP1 signaling pathways in vitro. To address the molecular mechanism by which DMP1 or mutant DMP1 regulate cell signaling, we compared their efficiency in activation of MAPK signaling in an odontoblast cell line in vitro.

**Fig. 4:**
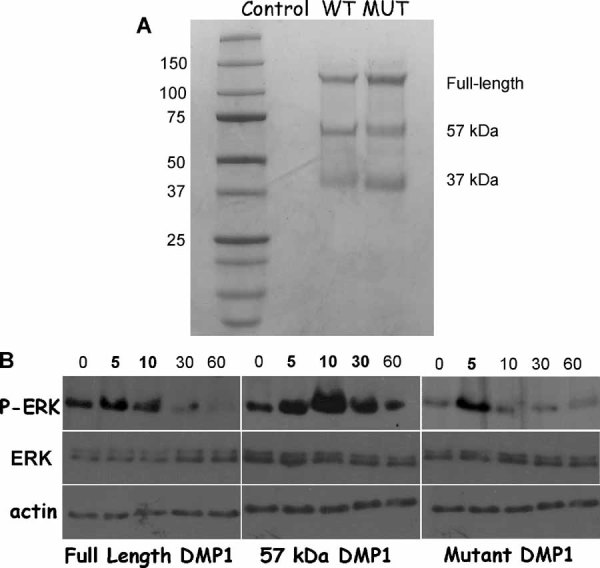
The recombinant delMut protein retains a partial function of DMP1 in activation of MAPK signaling. (*A*) The wild type (WT) recombinant DMP1 and the deletion mutant (MUT) proteins, collected from the conditional medium, are cleaved in vitro with a stains-all assay. (*B*) Activation of phosphorylated ERK by full-length DMP1 (lasting for 10 minutes), the C-terminal 57-kDa DMP1 fragment (lasting for over 30 minutes), or the MUT DMP1 (lasting for 5 minutes only) with both nonphosphorylated ERK and actin as internal controls.

First, we generated the following three recombinant DMP1s: full-length DMP1, 57-kDa C-terminal fragment DMP1, and delMut DMP1. The stains-all assay showed that these recombinant proteins were secreted into the medium and that both the delMut DMP1 and WT DMP1 were cleaved in vitro (Fig. 4*A*). Next, we tested their efficiency in activation of MAPK signaling in an odontoblast cell line.(22) As shown in Fig. 4*B*, all these recombinant proteins activated phosphorylated ERK. However, the effect of the 57-kDa fragment lasted for over 30 minutes, the full-length protein effect lasted for approximately 10 minutes, and the delMut protein effect lasted for only 5 minutes (see “Discussion” for details).

### Odontoblast cells play a role in regulation of FGF-23 expression in vivo

**Fig. 5:**
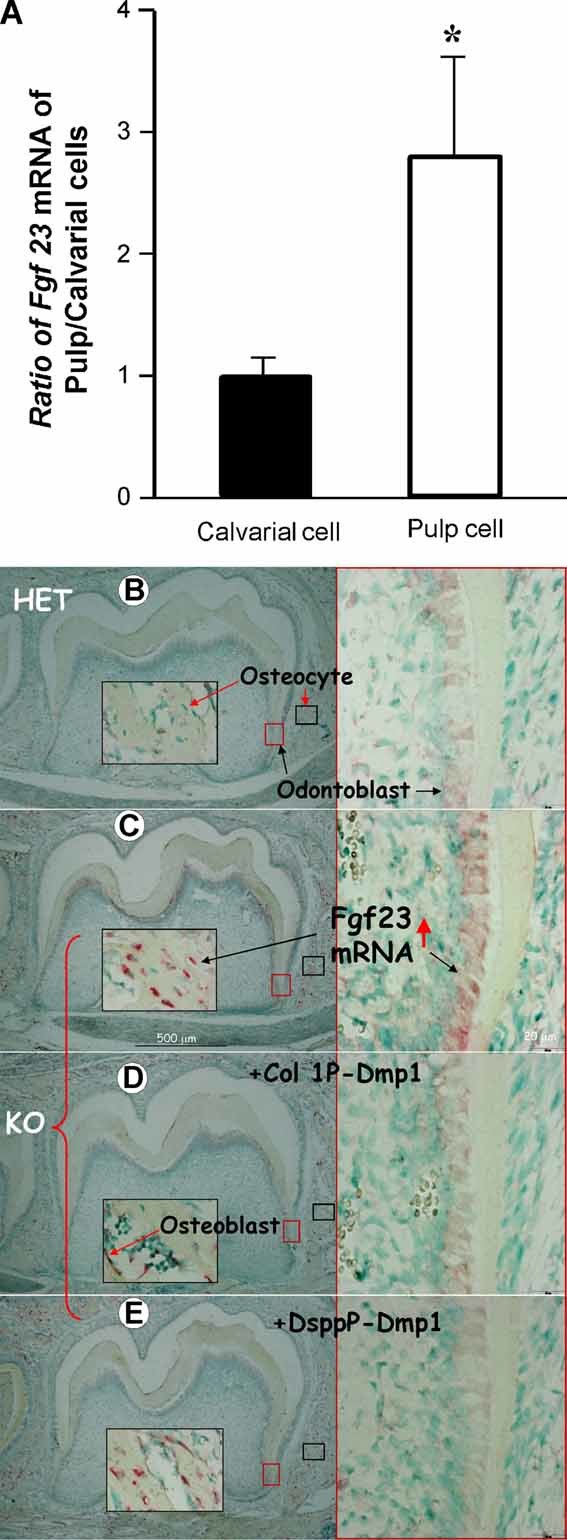
FGF-23 is higher in pulp/odontoblast cells than in bone cells. (*A*) *Fgf23* is expressed in newborn pulp/odontoblast cells higher than in bone cells, as shown by real-time RT-PCR. (*B*) *Fgf23* is expressed in control odontoblast cells (*signal in red*) at a much higher level than in bone, as shown by an in situ hybridization assay. (*C*) A sharp increase in both *Dmp1* KO osteocyte and odontoblast cells. (*D, E*) Changes in *Fgf23* mRNA in *Dmp1-*null osteocytes and odontoblast cells, where the murine *Dmp1* cDNA mouse is reexpressed under control of the 3.6-kb *Col1a1* promoter (for both bone cells and odontoblast cells, *D*) or under control of the *Dspp* promoter (for expression in odontoblast cells only, *E*). *Fgf23* mRNA levels are reduced in both KO odontoblast cells, where DMP1 is reexpressed (*D, E*). Note that *Fgf23* mRNA level is reduced only in the *Col1a1* DMP1 bone cells but not in the *Dspp* DMP1 bone cells.

FGF-23 has been demonstrated to be a critical physiologic and pathophysiologic factor in control of phosphate homeostasis in normal and several hypophosphatemic diseases.(24,25) Previously, we showed that FGF-23 is expressed mainly in osteoblasts in normal bone and sharply increased in *Dmp1-*null osteocytes.(12) To address whether the odontoblast cells play a role in FGF-23 regulation, we first compared *Fgf23* mRNA levels in newborn primary calvarial cells and pulp cells using real-time RT-PCR and showed a roughly 3-fold higher level in pulp cells than in calvarial cells (Fig. 5*A*). Next, we tested the expression pattern of *Fgf23* mRNA in 10-day-old odontoblasts using an in situ hybridization assay. As shown in Fig. 5*B*, the basal level of *Fgf23* mRNA in control odontoblast cells is much higher than that in bone cells. Second, we showed that the level of *Fgf23* mRNA was sharply increased in both *Dmp1-*null osteocytes and odontoblasts, although the changes in the *Dmp1-*null osteocytes were more striking than those in the *Dmp1-*null odontoblasts (Fig. 5*C*). Third, we retargeted expression of WT *Dmp1* in both null osteoblast/osteocyte and odontoblast cells by crossing transgenic mice harboring *Dmp1* driven by the 3.6-kb *Col1a1* promoter to *Dmp1-*null mice (Fig. 5*D*). The level of *Fgf23* mRNA was returned to the control level in the rescued cells. Lastly, retargeting *Dmp1* in the *Dmp1-*null odontoblast cell by the *Dspp* promoter that is activated mainly in odontoblast cells reduced the *Fgf23* level in the *Dmp1-*null tooth cell but not in the null bone cell (Fig. 5*E*). Taken together, this information suggests that odontoblast cells may play in control of FGF-23 level in a pathologic condition. However, this role might be very limited in comparison with the role of osteocytes.(12)

### Loss or overexpression of *Dmp1* has no effects on expressions of *Phex* mRNA in odontoblast cells in vivo

**Fig. 6:**
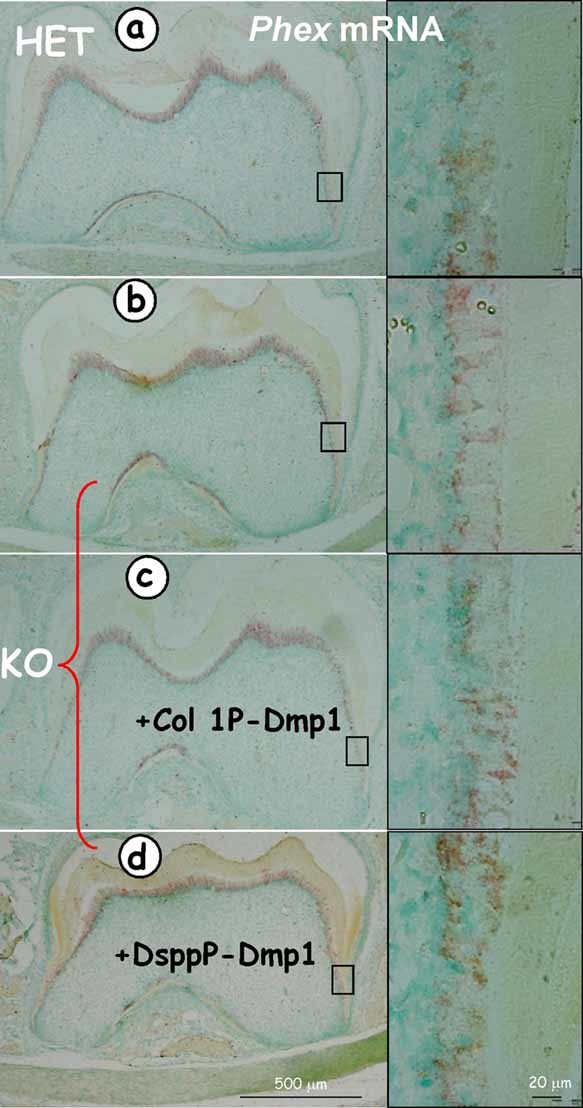
Loss of DMP1 or targeted reexpression of DMP1 has little effects on *Phex* expression in odontoblast cells. (*A*) *Phex* is expressed in control odontoblast cells (*signal in red*), as shown by an in situ hybridization assay. (*B–D*) Deletion of *DMP1* (*B*) or reexpression of *DMP1* (*C, D*) has little effects on *Phex* expressions in odontoblast cells.

Both *Dmp1* and *Phex* are coexpressed in bone and tooth. Deletions of either gene in mice or mutations of *DMP1* or *PHEX* in human patients display an identical hypophosphatemic rickets phenotype.(9,10,24,26) It has long been thought that there might be interactions between PHEX and DMP1 in some manner. To address this issue, we first confirmed *Phex* mRNA expression in odontoblast cells (Fig. 6*A*). Next, we analyzed *Phex* expression levels in *Dmp1-*null odontoblast cells with or without targeted reexpression of *Dmp1* in the *Dmp1-*null odontoblasts. Our results showed that *Phex* mRNA levels were largely unchanged, excluding the effects of *Dmp1* on *Phex* expression in the odontoblast cell (Fig. 6*B–D*).

## Discussion

Here we have used a clinical case and loss, gain, and mutant animal models, as well as a combination of X-ray, µCT, backscattered SEM, in situ hybridization, cell culture, and Western blot assays, to gain insight into the roles of DMP1 in tooth development. We find that (1) the delMut patient displayed enlarged pulp/root canals, thin dentin, and unexpected defects in enamel formation, which is similar to a dentinogenesis imperfecta III–like phenotype (see below for detail discussion), (2) the targeted expression of the delMut transgene, identical to that discovered in this patient, in the *Dmp1-*null background lead to a similar but slightly milder tooth phenotype. as observed in *Dmp1-*null mice, (3) mild changes are seen in serum FGF-23 and P_*i*_ levels in delMut mice, (4) *Fgf23* mRNA is expressed in control odontoblast cells with a much higher basal level than that in bone cells, (5) *Fgf23* mRNA in *Dmp1-*null odontoblast cells is upregulated, and reexpression of DMP1 in these *Dmp1-*null cells restores its original level, and (6) the delMut recombinant DMP1 is able to activate MAPK signaling but much less potently than WT DMP1. Overall, these results suggest that the delMut molecule remains a partial DMP1 function. In addition, the DI III–like phenotype observed in the delMut patient may be due to a second mutation or an as-yet-undetermined genetic modifier closely linked to DMP1 or related to the underlying dentin defect itself. The latter explanation is supported by the fact that abnormal dentin mineralization impairs development of the dentin enamel junction, which subsequently can affect enamel formation. Our data also support a hypothesis that odontoblast cells play a role in control FGF-23 regulation during early postnatal development.

Currently, there are two ways to produce a mutant mouse model. The first approach is to generate the targeted mutation to the locus where the endogenous *Dmp1* is located by using a classic homologous recombination technique. The second option is to overexpress the mutant *Dmp1*, under the control of a promoter highly active in bone and dentin, in a wild-type background or in the *Dmp1-*null background. We chose the latter based on the following: (1) DMP1 is expressed in both hard and soft tissues during developmental processes,(7,16,27,28) and targeting the mutant gene in hard tissue would fit our goal, (2) we have already generated and partially characterized *Dmp1-*null mice, and generation of transgenic mouse lines harboring the C-terminal mutant (Fig. 1*B*) would be straightforward with little risk, (3) we have previously successfully rescued the *Dmp1-*null phenotypes using the full-length *Dmp1* cDNA under the control of the *Col1a1* promoter,(18) which has the proper spatial-temporal characteristics to overexpress this gene in hard tissues, (4) varieties in *DMP1* mutations identified from ARHR patients or the DMP1 molecule with different mutations can be easily adapted into this system by generation of new transgenic mice, and (5) the first option, targeted mutation to the locus where endogenous *Dmp1* is located, has much higher risk partly owing to difficulties in homologous recombination and in getting the targeted cell into the germ line.

A significant question raised in this study is why the 1484–1490del mutation leads to much more severe dentin defects plus an enamel phenotype that is not observed in *Dmp1-*null mice. Based on our research results and the current literature (listed below), we propose that the deletion *DMP1* mutation contributes only to part of the dentin phenotype and that there might be another mutation or an as-yet-undetermined acquired gene modifier or other factor. First, the same mutant gene in the *Dmp1-*null background displays a mild dentin phenotype without an apparent defect in enamel (Fig. 2). It is of note that the deletion mutant molecule remains part of DMP1 function because this molecule still can activate MAPK signaling, although the effect is less potent (Fig. 4*B*). Second, even the homozygous *Dmp1-*null mice exhibit only a dentin phenotype with no changes in enamel.(10,29) Third, the heterozygous alleles of the human *DMP1* mutants (or *Dmp1-*null mice) or overexpression of this *Dmp1* deletion mutant in the WT background display no apparently abnormal dentin phenotype, excluding a dominant-negative effect on tooth (data not shown). In fact, other deletion mutant individuals from the same family display no DI III–like phenotype. Note that this deletion mutation patient manifested hypocalcemia in infancy that was suspected to have resulted from vitamin D deficiency. This nutritional deficiency was treated effectively. Whether the early nutritional deficiency affected enamel development in this patient remains unknown. Lastly, an ARHP kindred family with three affected individuals, in which a novel homozygous frame-shift mutation (c.485Tdel; p.Glu163ArgfsX53) in exon 6 led to a premature stop codon, was reported(14). These three patients showed a mild dentin defect with little change in enamel. These data are in agreement with our mutant mouse data.

The next question is, What caused the dentinogenesis imperfecta III–like phenotype in the delMut patient (Fig. 2*D*)? It is known that the most common gene mutation that leads to dentinogenesis imperfecta is the *DSPP* gene, whose mutations could lead to a dominant dentinogenesis imperfecta II or III phenotype.(30–33) However, we do not believe that a *DSPP* gene mutation is likely involved because other ARHR individuals do not show such severe tooth phenotypes. Our current speculation is that the *DMP1* deletion mutation triggers changes in the adjacent locus that result in an as-yet-undetermined acquired gene modification. This patient also had vitamin D deficiency in infancy, as discussed, which may have had an effect.

Another possibility for the phenotype difference is that it is due to species differences between humans and mice. One example is *DSSP* mutations. In humans, heterozygous *DSPP* mutations display a dominant pathologic phenotype such as a dentinogenesis imperfecta syndrome.(30–33) On the other hand, there is no apparent phenotype in heterozygous *Dspp-*null mice, and the dentin defects occur only in homozygous mice.(34)

There is also a spatial-temporal difference in the endogenous *DMP1* promoter activity necessarily functional in the human population compared with the exogenous, randomly inserted *Col1a1*-mutant *Dmp1* transgene used in mice. In other words, we chose a transgenic animal approach (rather than a targeted knock-in) and used the *Col1a1* promoter (rather than a *Dmp1* promoter), and the promoter activity is necessarily different in these mutant animals. The targeted-insertion approach was not used here but may have given a phenotype in mice more closely resembling that of the human population. The dentin abnormalities themselves may affect enamel formation, yet another potential cause for the abnormal enamel findings in this patient.

A final intriguing issue is that expressions of FGF-23 (a newly discovered factor for control of phosphate reabsorption in the kidney) in odontoblast cells may contribute to phosphate homeostasis regulation during early postnatal development. It is well documented that FGF-23 is released mainly from bone cells.(24) Here, both our in vitro and in vivo data showed that the level of *Fgf23* mRNA in odontoblasts is much higher than that in bone cells (Fig. 5*A*). Note that mouse molars are fully mature at the age of approximately 8 weeks, when the odontoblast cells are no longer active. This may explain in part why the serum level of FGF-23 is higher (∼350 pg/mL) in 2-week-old pups than in 7-week-old mice (∼100 pg/mL; Dr. Baozhi Yuan, University of Wisconsin–Madison, personal communication). Furthermore, loss of *Dmp1* leads to an upregulation of *Fgf23*, and overexpression of *Dmp1* in the null background results in downregulation of *Fgf23* (Fig. 5*B–D*). Taken together, these findings support a hypothesis that the odontoblast cell, as an endocrine cell, may contribute to FGF-23 regulation during early postnatal development. However, the odontoblast mass is much smaller than that of bone cells. This regulation of P_*i*_ homeostasis is likely very limited.

In summary, the successful generation and characterization of a ARHR delMut model provides a powerful tool for studies of the pathophysiologic process of this disease. Our initial study suggests that *DMP1* mutations are responsible for the defects in dentinogenesis and that the severe defect in both dentin and enamel in the delMut patient is likely due to an as-yet-undetermined acquired gene modifier or other unidentified factor. Our studies on expression patterns of FGF-23 in odontoblast cells support a new notion that odontoblasts participate in control of FGF-23 expression during early postnatal development.
