# Supplementary material for: DMP1 C-Terminal Mutant Mice Recapture the Human ARHR Tooth Phenotype
Source: J Bone Miner Res. 2010 Apr 30;25(10):2155–64. doi: 10.1002/jbmr.117 (PMC3153318; doi:10.1002/jbmr.117)
Supplement: Supplementary file 1 [file jbmr0025-2155-SD1.pdf]

Supplementary data

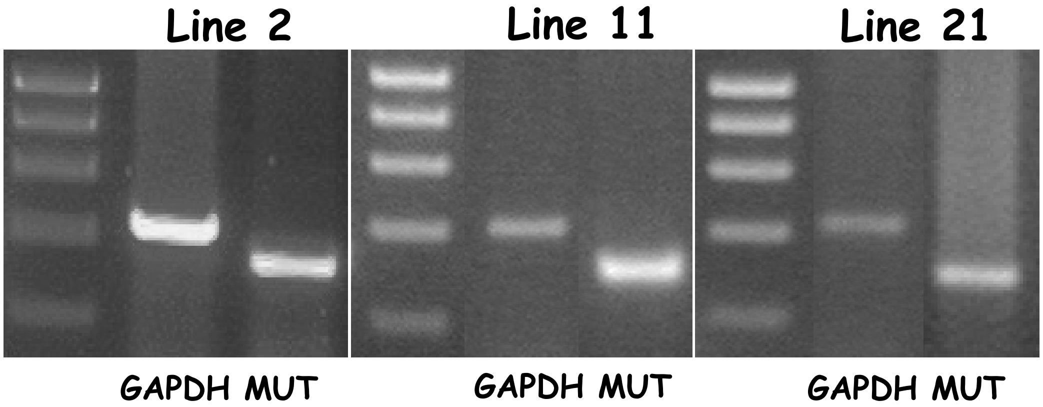

Fig. S1 Expression of the *Dmp1* mutant transgene in vertebrae by RT-PCR. Three out of 5 independent transgenic lines (lines 2, 11 and 21) were initially used for expression analysis by RT-PCR (see “Materials and Methods”). The sequences of the primers for mutant *Dmp1* transgene were: 5′-CAGCCGTTCTGAG GAAGACAGTG-3′ (from *Dmp1* cDNA) and 5′-TGTCCAAACTCATCAATGTATCT-3′ (from SV40 polyadenylation signal peptide); and the primer sequences for Gapdh were 5′-GGTGTGAACCACGAGAAATA-3′ and 5′-TGAAGTCGCAGGAGACAACC-3′ with 35 cycles.
